# Higenamine Attenuates Doxorubicin-Induced Cardiac Remodeling and Myocyte Apoptosis by Suppressing AMPK Activation

**DOI:** 10.3389/fcell.2022.809996

**Published:** 2022-05-05

**Authors:** Cuiliu Jin, Yu Chai, Zhimin Hu, Wencong Tian, Wang Ling, Jing Li, Meiping Wu

**Affiliations:** ^1^ Department of Cardiology, Shanghai Municipal Hospital of Traditional Chinese Medicine, Shanghai University of Traditional Chinese Medicine, Shanghai, China; ^2^ Department of Molecular Pharmacology, School of Medicine, Nankai University, Tianjin, China

**Keywords:** doxorubicin, higenamine, adenosine-activated protein kinase, cardiac remodeling, myocyte apoptosis

## Abstract

**Background:** As an effective antitumor drug, doxorubicin (DOX) is primarily used to treat solid tumors and hematologic malignancies. However, increasing evidence has emerged indicating its cardiotoxicity, and few solutions have been proposed to counter this side effect. Higenamine (HG) is a natural compound widely found in many Chinese herbs and also serves as a component in many healthcare products. Several studies have demonstrated its cardioprotective effect in different models, but little is known about the underlying influences of HG against myocardial damage from DOX-induced chronic cardiotoxicity.

**Methods and Results:** C57BL/6 mice and neonatal rat ventricular cardiomyocytes (NRVMs) were used to evaluate the cardioprotective effect of HG against DOX-induced myocardial damage. In mice, DOX (intraperitoneally injected 5 mg/kg every 3 days for 4 weeks) significantly increased cardiomyocyte apoptosis, cardiac atrophy, and cardiac dysfunction, which were significantly attenuated by HG (intragastrically administered with 10 mg/kg every day for 4 weeks). In NRVMs, DOX (3 μM for 24 h) significantly increased cell apoptosis and the level of reactive oxygen species while reducing the level of superoxide dismutase and mitochondrial membrane potential. Remarkably, HG can reverse these pathological changes caused by DOX. Interestingly, the protective effect of HG on DOX-induced cardiotoxicity was independent of the activation of the beta-2 adrenergic receptor (β2-AR), known for mediating the effect of HG on antagonizing ischemia/reperfusion-induced cardiac apoptosis. Furthermore, HG attenuated the abnormal activation of phosphorylated adenosine-activated protein kinase (AMPK). Consistently, AMPK agonists (AICAR) can eliminate these pharmacological actions of HG.

**Conclusion:** Collectively, our results suggested that HG alleviated DOX-induced chronic myocardial injury by suppressing AMPK activation and ROS production.

## Introduction

Tumor cardiopathy is a major disease that affects the survival rate and time of tumor patients, where chemotherapy-induced cardiotoxicity accounts for significant complications (Koutsoukis et al., 2018). Doxorubicin (DOX), a cornerstone of chemotherapy, is widely known for its cardiotoxicity, which is difficult to combat in the clinic (Singal and Iliskovic., 1998). DOX-induced cardiomyocyte apoptosis, myocardial atrophy, dilated cardiomyopathy, and myocardial fibrosis contribute to myocardial remodeling, left ventricular dysfunction, and even heart failure (Zhang et al., 2018; Galan-Arriola et al., 2019).

At present, the mechanism of cardiotoxicity induced by DOX is not fully understood. Increasing research has indicated that it might be a multifactorial process including oxidative stress, inflammatory response, mitochondrial damage, disorder of calcium metabolism, induced myocardial cell apoptosis, and autophagy (Mantawy et al., 2014; Wang et al., 2021). Moreover, most drugs which can protect heart remodeling in other types of heart disease fail to do so with DOX-induced cardiotoxicity. The exception to this is dexrazoxane (Yu et al., 2020), the only drug authorized by the FDA to reduce the incidence and severity of cardiomyopathy associated with DOX administration. Furthermore, the administration of dexrazoxane is not 100% effective, so seeking new therapies for the treatment of cardiotoxicity has garnered significant attention. As the primary component of the heart structure, cardiomyocytes are the basis to maintain normal physiological functions of the heart. The fate of cardiomyocytes from DOX treatment has attracted significant attention from the bench to the clinic (Hagag et al., 2020).

Higenamine {HG; 1-[(4-hydroxyphenyl)methyl]-1,2,3,4-tetrahydroisoquinoline-6,7-diol} is the main active ingredient of aconite root, a traditional Chinese herb which was widely used to treat heart failure-like symptoms in Asian countries for thousands of years. Interestingly, HG has been also used as a common component of health products for fat degradation and sports performance in Europe and North America (Lee et al., 2013; Liu and Santillo., 2016). In recent years, a vast number of clinical experimental studies have proven that HG can serve as a cardiotonic, tracheal smooth muscle relaxant, anticoagulant, and anti-inflammatory agent (Tsukiyama et al., 2009; Zhang et al., 2014; Tariq and Aronow, 2015).

Our previous studies have reported that the β2/PI3K/Akt signaling pathway can be activated by HG to inhibit the myocardial injury induced by ischemia-reperfusion (Wu et al., 2016). It has also been found that HG can inhibit myocardial injury and cardiac fibrosis *via* the TGF-β/Smad signaling pathway, which inhibits the activation of cardiac fibroblasts and reduces the deposition of matrix proteins (Zhu et al., 2021). Although these studies have confirmed the therapeutic effect of HG on cardiovascular diseases, it is uncertain whether HG can protect against DOX-induced cardiotoxicity. In the current study, we have examined the effects of HG in chronic DOX-induced heart injury in C57 mice. Using NRVMs, we examined the effects of HG on NRVM apoptosis and the levels of ROS induced by DOX. We also determined the mechanism of HG in this pharmacological function. Here, we have demonstrated that HG remarkably inhibits myocardial apoptosis induced by DOX and restores heart functions. Furthermore, we found that the mechanism of HG in these diseases is different from known pathways, partially through suppressing the abnormal AMPK activation and ROS production, which has not been reported yet.

## Materials and Methods

### Reagents and Drugs

HG was purchased from TAUTO Biochemical Technology Co., Ltd. (Shanghai, China). DOX was procured from MedChemExpress (Shanghai, China). The primary antibodies for cleaved caspase 3, GAPDH, phosphor-AMPKα, and total-AMPKα were purchased from Cell Signaling Technology (Danvers, MA, United States). Cell culture media were purchased from Gibco (Grand Island, NY, United States).

### Animal Care

Adult male C57BL/6 mice, 8 weeks of age, were purchased from Shanghai Slac Laboratory Animal Co. Ltd. The mice were routinely kept in the animal room of Shanghai Tongji University. The protocols were approved by the Laboratory Animal Ethics Committee of Shanghai Tongji University (Shanghai, China; Permit No. TJHBLAC-2019-057). All animal procedures were performed in accordance with the Shanghai University of Traditional Chinese Medicine guidelines and the National Natural Science Foundation of China (NSFC).

### Doxorubicin Injection Model

**FIGURE 1:**
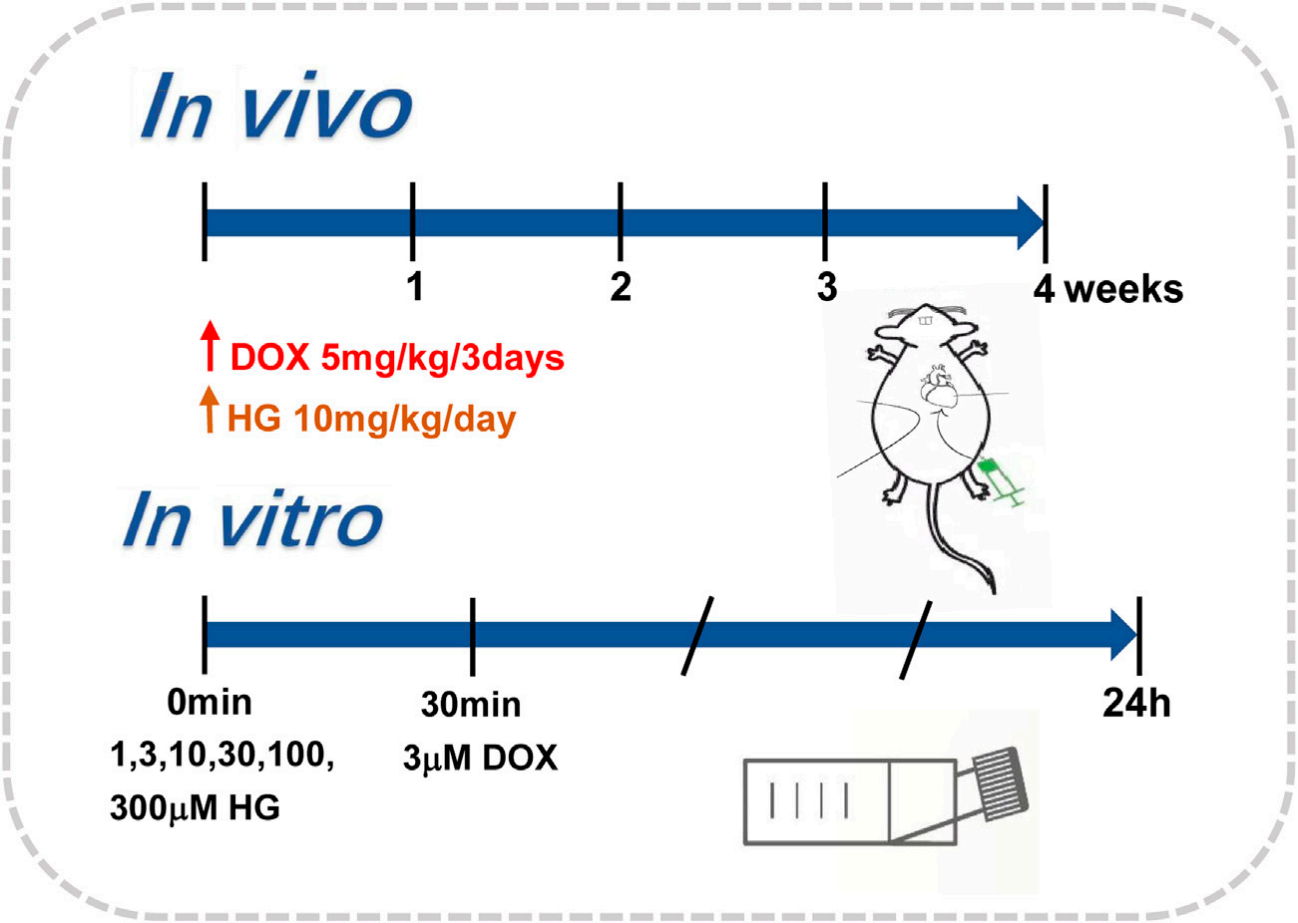
Administration modes *in vivo* and *in vitro*.

To mimic chronic cardiotoxicity of DOX *in vivo*, we exposed the mice to continuous intraperitoneal injection of vehicle saline or DOX. The mice in DOX and DOX + HG groups were intraperitoneally injected with 5 mg/kg DOX every 3 days for 4 weeks. For HG treatment, HG was dissolved in a vehicle consisting of 10% (vol/vol) DMSO in 40% β-cyclodextrin. The mice were then randomized to receive either HG (10 mg/kg) or vehicle (10% DMSO in 40% β-cyclodextrin) treatment administered intragastrically once daily for 4 weeks. The aforementioned procedure is described in Figure 1. After 4 weeks, the general condition and survival of the mice were observed.

### Neonatal Rat Ventricular Cardiomyocytes Isolation, Culture, and Treatment

NRVMs were isolated from 2- to 3-day-old suckling Sprague Dawley rats following the methods previously published (Golden et al., 2012). In brief, the rat hearts were excised; their ventricles were separated and washed in Hank’s balanced salt solution (HBSS) three times and then digested repeatedly with type II collagenase containing HBSS. The cells were collected by 100 × g centrifugation and resuspended in Dulbecco’s modified Eagle medium (DMEM), supplemented with 15% fetal bovine serum (FBS), 100 IU/ml penicillin, and 100 g/ml streptomycin. Fibroblasts were then removed by differential adherent culture and placed at 37°C for 90 min. The cell suspension was collected and plated directly into six-well plates with 5-BrdU in a density of about 3 × 10^5^ cells/ml. After 24 h, the cells were washed several times with PBS and treated with DMEM containing 10% fetal bovine serum. Cardiomyocytes were treated with DOX (3 μM) for 24 h, with or without different interventions. The aforementioned procedure is described in Figure 1.

### Echocardiography

Cardiac function was assessed by transthoracic echocardiography and using a Vevo 2100 ultra-high resolution small animal ultrasound imaging system (Fujifilm, Toronto, ON, Canada). Left ventricular systolic and diastolic functions, LV ejection fraction (EF%), and fractional shortening (FS%) were measured.

### Assessment of Cell Viability

Cell viability was measured by using the Cell Counting Kit-8 (CCK-8). In brief, after 24 h of treatment, cells were incubated with CCK-8 solution (Beyotime, China). To facilitate the reaction, stained cells were incubated at 37°C for 3 h. At the end of the treatment, the absorbance at 450 nm was measured using a BioTek^®^ Epoch microplate reader (BioTek Instruments, Inc. United States).

### Assessment of Reactive Oxygen Species Levels

Intracellular reactive oxygen species levels were assessed by using a DCFH-DA fluorescence probe (Beyotime, China). In brief, after 24-h treatment, cells were incubated with a 10 μM DCFH-DA fluorescence probe for 30 min at 37°C in the dark. The fluorescence intensity was determined by flow cytometry.

### Assessment of Superoxide Dismutase Levels

Intracellular superoxide dismutase levels were measured by using superoxide dismutase kits (Beyotime, China), following the manufacturer’s instructions. The data were measured by using a BioTek^®^ Epoch microplate reader (BioTek Instruments, Inc. United States).

### Western Blot Analysis

Western blot analysis was performed as described previously (Knight et al., 2016). In brief, cell and heart samples after various treatments were collected to extract the proteins. The protein concentration was determined by using a BCA protein assay (Thermo Fisher Scientific, Rockford, IL, United States). The protein samples were separated by SDS-PAGE and transferred to PVDF membranes. PBST containing 5% (m/v) BSA (in Tris buffer containing 0.1% Tween 20) was used to block the membrane at room temperature for 2 h. The cells were then incubated with anti-AMPK (1:1000), anti-P-AMPK (THR 172, 1:1000), anti-cleaved caspase 3 (1:1000), and anti-GAPDH (1:1000) primary antibodies at 4°C overnight. Subsequently, the membrane was washed with PBST and exposed to the corresponding secondary antibody (1:6000) at room temperature for 2 h. A Bio-Rad imaging system (Bio-Rad, Hercules, CA, United States) was used to detect fluorescence signals, and Image Lab software (Bio-Rad, Hercules, CA, United States) was used to quantify the signal.

### Hematoxylin–Eosin Staining

Hematoxylin–eosin staining was performed as described previously (Cardiff et al., 2014). Mice hearts were collected, fixed, embedded, and sectioned, in accordance with the standard protocol. In short, the heart sections were dewaxed with xylene and dehydrated with ethanol. The sections were incubated in the hematoxylin staining solution at room temperature for 5–10 min, washed with distilled water for 5 min, re-stained with eosin staining solution for 3 min, and finally washed with distilled water for 5 min. The sections were soaked in 75, 85, 95, and 100% ethanol for 2 min, and then heart sections were cleaned in xylene and sealed with neutral resin.

### Wheat Germ Agglutinin Staining

Wheat germ agglutinin (WGA) staining was performed as described previously (Wang et al., 2019). In brief, heart sections were dewaxed with xylene and dehydrated with ethanol. The heart tissues were preheated in a pressure cooker containing 0.01 M citrate solution, incubated with glycine at room temperature for 20 min, and washed with PBS for 5 min. WGA staining solution was prepared at a dilution of 1:50. The staining solution was dropped on the glass slide and incubated at room temperature for 1 h in the dark. The slides were then washed three times with PBS, taking 5 min per wash. DAPI sealing solution was then added to seal the heart sections. Images were collected by using a fluorescence microscope. Image-Pro software (Media Cybernetics) was used to quantify the mean cardiomyocyte cross-sectional area (CSA).

### Terminal Deoxynucleotidyl Transferase–Mediated dUTP Nick-End Labeling Staining

TUNEL staining was performed in accordance with the instructions of the TUNEL kit (Roche, Switzerland). In brief, the tissue sections were dewaxed and rehydrated following standard protocols. The tissue sections were incubated for 30 min at 37°C with proteinase K working solution (20 μg/ml in 10 mM Tris–HCl, pH 7.4–8). The blocking solution (5% goat serum in PBS) was then added and incubated for 1 h at room temperature. The TUNEL reagent was added to the samples and incubated at 37°C for 1 h in the dark. After washing with distilled water three times (5 min each), DAPI dye solution was added and incubated at room temperature for 10 min. After washing with distilled water twice, images were taken under a fluorescence microscope. The images were obtained from three random regions of each mouse heart section. The apoptotic index was expressed as the percentage of TUNEL-positive nuclei to DAPI-stained nuclei.

### Measurement of Mitochondrial Membrane Potential

The mitochondrial membrane potential (MMP) was evaluated by staining with a cationic dye, 5,5,6,6-tetrachloro-1,1,3,3-tetraethylbenzimidazolylcarbocyanine iodide (JC-1, Beyotine, China), in accordance with the manufacturer’s instructions. In brief, neonatal rat cardiomyocytes were inoculated into six-well plates; the culture medium was aspirated, and the cells were washed with PBS. Afterward, 1 ml of the culture medium and 1 ml of the JC-1 staining solution were added and the mixture was incubated at 37°C for 20 min. After incubation, the cells were washed twice with 1X buffer and the culture medium was added. The images were normalized by fluorescence imaging using a Leica inverted fluorescence microscope.

### Immunohistochemistry

For immunohistochemistry, the heart paraffin sections were heated using the pressure cooker for antigen retrieval and 8% goat serum was used to block non-specific binding sites incubated with anti-P-AMPK (ab23875, Abcam) and anti-T-AMPK antibody (ab131512, Abcam), followed by incubation with goat anti-rabbit EnVisionTM+/horseradish peroxidase (HRP) reagent, and stained using a DAB detection kit (GeneTech, Shanghai, China). Negative control was obtained by replacing primary antibody with PBS. Immunohistochemistry paraffin sections were visualized by light microscopy.

## Statistical Analysis

All data are presented as mean ± SEM. Statistical analysis was performed using Prism 8.0 one-way ANOVA, followed by Bonferroni’s post hoc test for comparisons of multiple groups. p-values < 0.05 were considered statistically significant.

## Results

### Higenamine Attenuates DOX-Induced Cardiac Injury *In Vivo*

**FIGURE 2:**
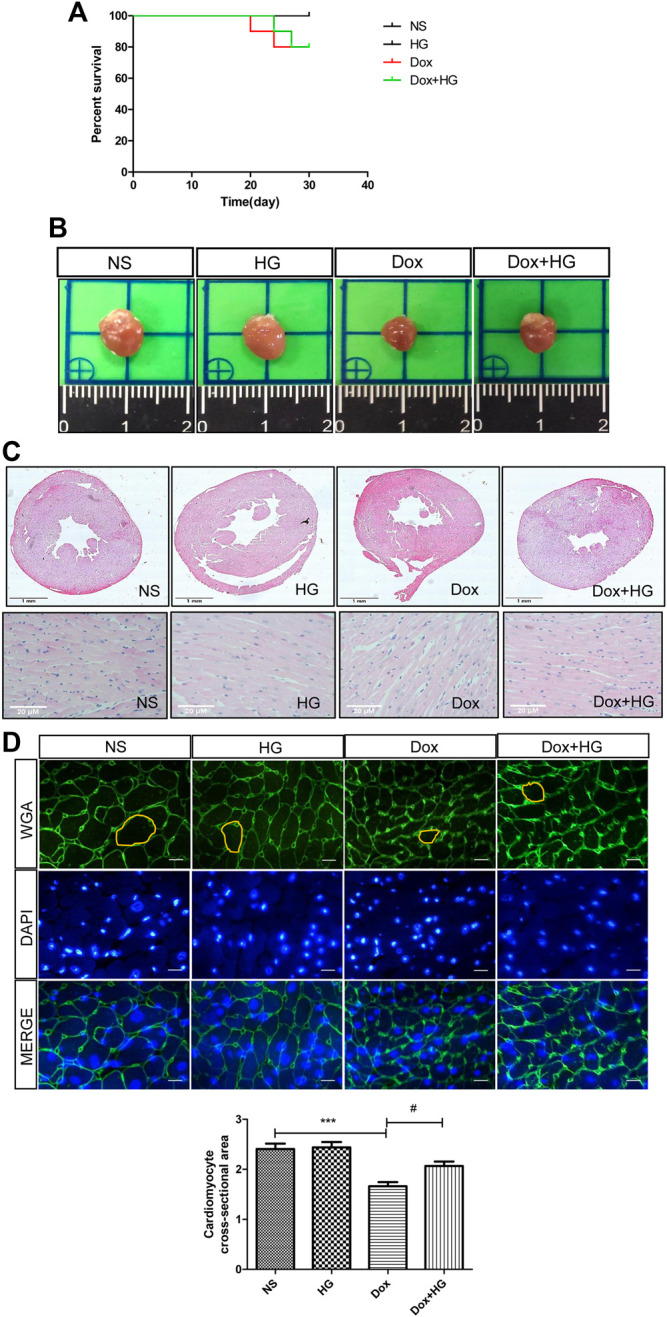
HG attenuated DOX-induced cardiac injury *in vivo*. **(A)** Survival curve of mice. **(B)** Representative heart images of each group. Mice were injected with normal saline (NS) or DOX (5 mg/kg/3 days) and systemically administrated with HG (10 mg/kg/day) during DOX injection for 4 weeks. **(C)** Schematic diagram of HE staining of the heart cross-sectional area for each group. **(D)** Schematic diagram of representative heart tissue WGA staining for each group of mice. **(E)** Quantification of the cross-sectional area of single cardiac muscle cells after WGA staining. All values are presented as mean ± SEM; statistical analysis was performed using Prism 8.0 one-way ANOVA. * *p* < 0.05 DOX vs NS; # *p* < 0.05 DOX + HG vs DOX. Animal numbers: NS, n = 8; HG, n = 7; DOX, n = 10; Dox + HG, n = 10.

To explore the function of Higenamine in cardiac injury induced by DOX *in vivo*, C57BL/6 mice were intraperitoneally injected with 5 mg/kg of DOX every 3 days for 4 weeks to mimic the chronic myocardial injury induced by DOX. For HG intervention, mice were randomized to receive either HG (10 mg/kg) or vehicle [10% (vol/vol) DMSO in 40% β-cyclodextrin] treatment administered intragastrically once daily starting from the first day of DOX injection. As shown in Figure 2A, after 4 weeks, there was no statistical difference in the survival rate among each group. DOX-induced heart atrophy was assessed by global morphology. Daily HG application reversed this pathological deterioration (Figure 2B). H&E staining was used to evaluate cardiac remodeling, and WGA staining was performed to illustrate the cross-sectional area of each cardiomyocyte. Using H&E staining, we found a similar tendency of morphological changes in the global shape (Figure 2C). Consistently, WGA staining indicated that HG can reverse the DOX-induced cross-sectional area (CSA) reduction of cardiomyocytes (Figures 2D,E), suggesting that HG attenuates DOX-induced cardiac remodeling *in vivo*.

### Higenamine Improves Doxorubicin-Induced Cardiac Dysfunction *In Vivo*

**FIGURE 3:**
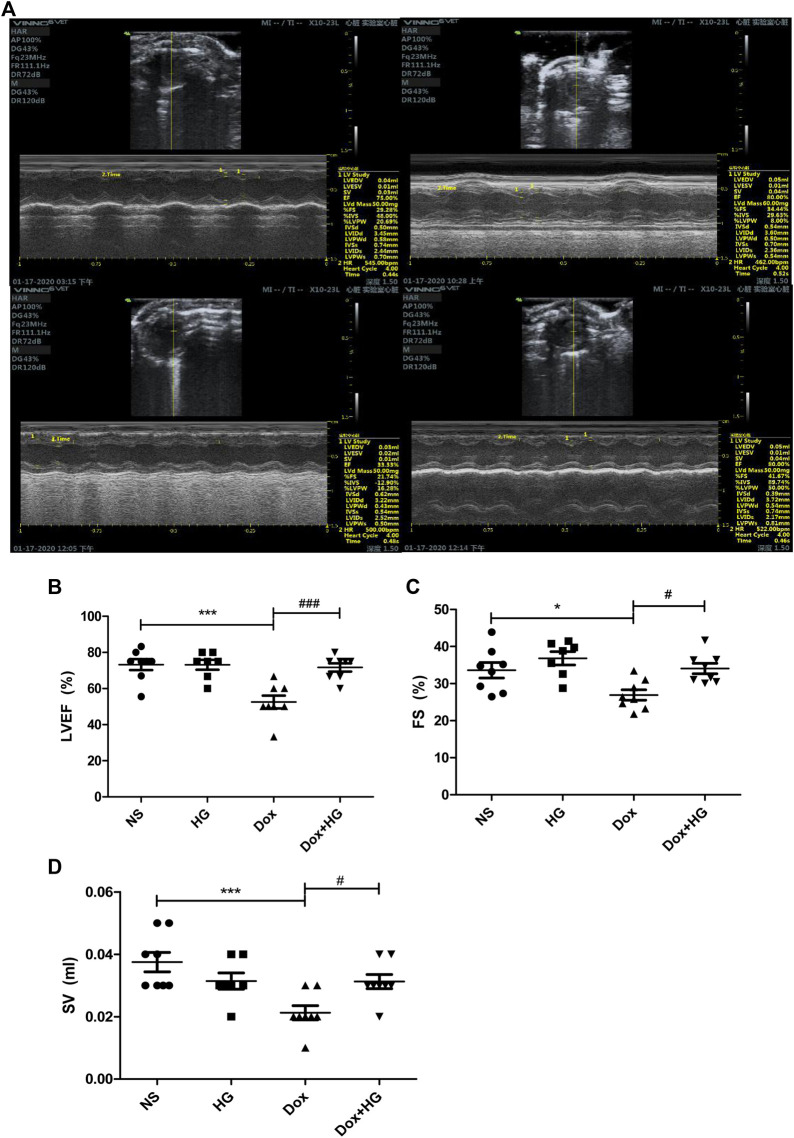
HG improved DOX-induced cardiac dysfunction *in vivo.* **(A)** Representative M-mode echocardiographic images of mice in each group at 4 weeks. **(B–D)** Summary of **(B)** ejection fraction (EF), **(C)** shortening fraction (FS), and **(D)** stroke volume (SV) data in each group. All values are presented as mean ± SEM; statistical analysis was performed using Prism 8.0 one-way ANOVA. * *p* < 0.05 DOX vs NS; # *p* < 0.05 DOX + HG vs DOX. Animal numbers: NS, n = 8; HG, n = 7; DOX, n = 10; DOX + HG, n = 10.

To evaluate the effects of HG on the cardiac dysfunction induced by DOX, we monitored cardiac function by echocardiography (Figure 3A and Table 1). As depicted in Figures 3B,C, compared with the normal saline group, the cardiac function in the DOX model group decreased, reflected by the ejection fraction (EF) and shortening fraction (FS) (*p* < 0.05). With HG application, EF and FS were significantly rescued (*p* < 0.05). Consistently, DOX induced heart atrophy as reflected by the stroke volume (SV), and this deterioration is markedly attenuated in mice receiving treatment with HG (*p* < 0.05) (Figure 3D). Using the ELISA kit to detect BNP levels in serum, the results showed that DOX can also promote BNP content in serum, and HG can reduce BNP content (Supplementary Figure S3A). All these results suggest that HG has a protective effect on cardiac remodeling and heart failure induced by DOX.

**TABLE 1:** Echocardiographic indexes in each group of mice.

Parameter	NS	HG	Dox	Dox + HG
EF (%)	73.195 ± 8.028	73.096 ± 6.751	52.5 ± 9.393*	71.668 ± 6.123#
FS (%)	33.606 ± 5.522	36.829 ± 4.379	26.875 ± 3.683*	34.073 ± 3.753#
LVEDV (ml)	0.053 ± 0.016	0.043 ± 0.007	0.04 ± 0.007	0.044 ± 0.009
LVESV (ml)	0.015 ± 0.010	0.011 ± 0.003	0.019 ± 0.003	0.013 ± 0.004
SV (ml)	0.038 ± 0.008	0.031 ± 0.006	0.021 ± 0.006*	0.031 ± 0.006#
IVSd (mm)	0.509 ± 0.101	0.461 ± 0.029	0.544 ± 0.074	0.509 ± 0.066
IVSs (mm)	0.7 ± 0.053	0.683 ± 0.085	0.635 ± 0.072	0.649 ± 0.107
LVIDs (mm)	2.495 ± 0.393	2.22 ± 0.171	2.519 ± 0.154	2.311 ± 0.219
LVIDd (mm)	3.744 ± 0.354	3.516 ± 0.160	3.445 ± 0.142	3.506 ± 0.259
LVPWs (mm)	0.709 ± 0.077	0.797 ± 0.098	0.639 ± 0.109	0.679 ± 0.070
LVPWd (mm)	0.586 ± 0.068	0.603 ± 0.070	0.543 ± 0.079	0.509 ± 0.054
HR(beats/min)	539.00 ± 37.964	510.714 ± 65.226	500.75 ± 31.52	482.625 ± 35.6

*p < 0.05 vs NS; #*p* < 0.05 vs Dox.

Values are expressed as mean ± SEM.

EF, ejection fraction; FS, fractional shortening; LVEDV, left ventricular end-diastolic volume; LVESV, left ventricular end-systolic volume; SV, stroke volume; IVSd, diastolic interventricular septal thickness; IVSs, systolic interventricular septal thickness; LVIDs, left ventricular internal diameter in systole; LVIDd, Left ventricular internal diameter in diastole; LVPWs, left ventricular posterior wall thickness at systole; LVPWd, left ventricular posterior wall thickness at diastole; HR, heart rate.

All values are presented as mean ± SEM; statistical analysis was performed using Prism 8.0 one-way ANOVA.

### Higenamine Inhibited Doxorubicin-Induced Cardiomyocyte Apoptosis *In Vivo*

**FIGURE 4:**
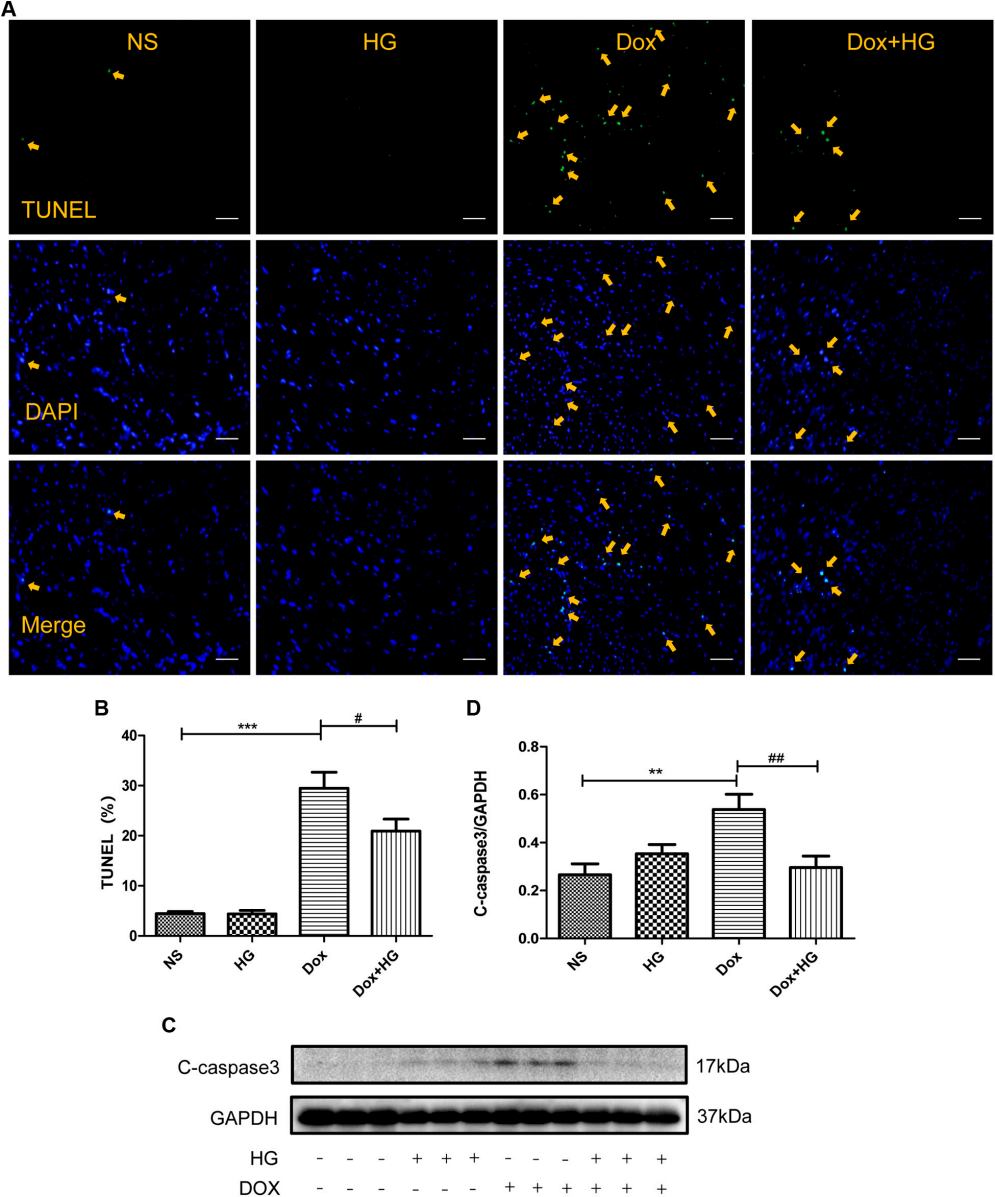
HG inhibited DOX-induced cardiomyocyte apoptosis *in vivo.* **(A)** Representative TUNEL staining image of a myocardial section. Nuclei of apoptosis cells are marked with orange arrows. **(B)** Quantitative data of nuclei of apoptosis cells in mice evaluated by TUNEL staining. **(C)** Western blot analysis of GAPDH, cleaved caspase 3 in cardiac tissues expressed in mice. **(D)** Quantitative data of cleaved caspase 3 expressed in mice. All values are presented as mean ± SEM; statistical analysis was performed using Prism 8.0 one-way ANOVA. * *p* < 0.05 DOX vs NS; # *p* < 0.05 DOX + HG vs DOX. Animal numbers: NS, n = 8; HG, n = 7; DOX, n = 10; DOX + HG, n = 10.

Cardiomyocyte apoptosis is a pivotal pathological process of DOX-induced myocardial injury. To evaluate the effect of HG on DOX-induced cardiomyocyte apoptosis, TUNEL staining and Western blots were performed on mice hearts. As expected, TUNEL staining showed that mice with DOX had a significantly increased number of apoptotic cardiomyocytes compared to the normal saline group, and HG could inhibit DOX-induced cardiomyocyte apoptosis (Figures 4A,B). Similarly, Western blot results showed that compared to the saline control mice, the expression level of cleaved caspase 3 in mice with DOX was largely increased (*p* < 0.05). After application with HG, the protein level of cleaved caspase 3 was significantly decreased (*p* < 0.05) (Figures 4C,D). Combined with the TUNEL staining, these results confirmed that HG could attenuate DOX-induced cardiac apoptosis in mice. Doxorubicin has been proved to lead to adverse ventricular remodeling of the heart in the chronic progressive approach, mainly involved in cardiac fibrosis. In order to detect the degree of myocardial fibrosis after DOX stimulation, we performed PSR staining and Masson staining. The results showed that myocardial fibrosis did not increase after injecting DOX for 4 weeks, and HG treatment did not remarkably reduce myocardial fibrosis and the perivascular collagen volume area (Supplementary Figures S1A–D).

### Higenamine Protected Doxorubicin-Induced Neonatal Rat Ventricular Myocyte Apoptosis *In Vitro*

**FIGURE 5:**
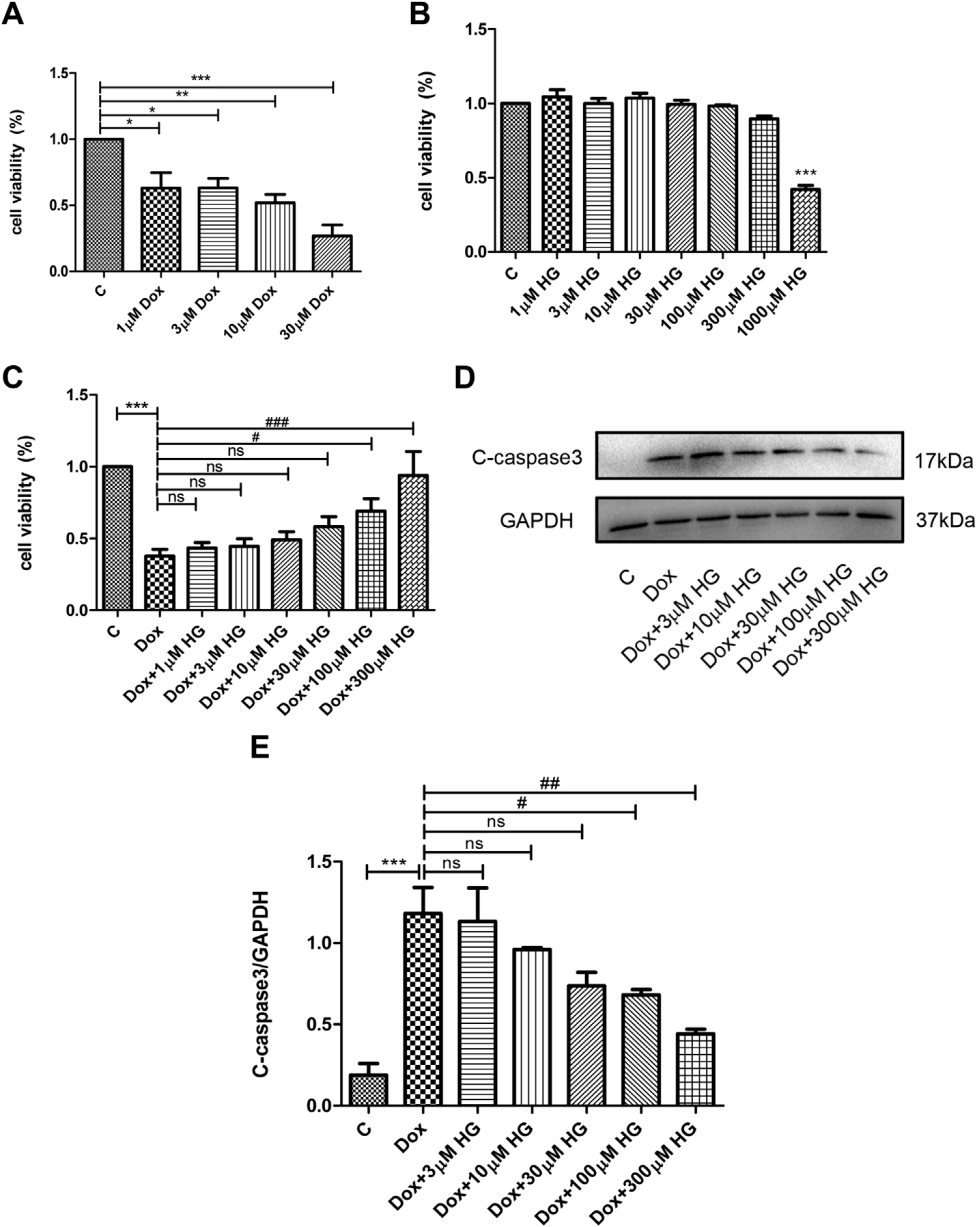
HG inhibited DOX-induced NRVM apoptosis *in vitro*. **(A)** NRVMs were treated with DOX (1, 3, 10, and 30 μM) for 24 h after detection by the CCK8 reagent. **(B)** NRVMs were treated with HG (1, 3, 10, 30, 100, 300, and 1000 μM) for 24 h after detection by the CCK8 reagent. **(C)** NRVMs were pretreated with HG (1, 3, 10, 30, and 100 μM) for 30 min after treatment with DOX for 24 h. Cell activity was detected by using the CCK8 reagent. **(D)** Protein level of cleaved caspase 3 was detected by Western blot. **(E)** Quantitative data of cleaved caspase 3 expressed in NRVMs. The aforementioned experiments were repeated more than three times. All values are presented as mean ± SEM; statistical analysis was performed using Prism 8.0 one-way ANOVA. * *p* < 0.05 DOX vs Control C and HG vs Control C; # *p* < 0.05 DOX + HG vs DOX; ns, *p* > 0.05 DOX + HG vs DOX.

Various concentrations of DOX were used to stimulate NRVMs for 24 h *in vitro*. As shown in Figure 5A, 1–3 μM DOX was enough to cause death of half of the NRVMs (*p* < 0.05). As the concentration of DOX increased, the proportion of NVRMs that died also increased. In order to further explore whether HG can inhibit DOX-induced cytotoxicity in NRVMs *in vitro*, we first tested cytotoxicity of HG. After incubation with various concentrations of HG for 24 h, the activity of NRVMs did not significantly decrease when the concentration of HG was less than 1 mM, indicating that HG itself has no obvious cytotoxic effect on NRVMs (Figure 5B). Based on the results depicted in Figure 5A, we used 3 μM DOX-induced NRVM death as the cell model and added varying concentrations of HG 30 min prior to DOX. The results showed that when the concentration of HG was under 30 μM, it could not effectively inhibit DOX-induced NRVM death (*p* > 0.05). However, when the concentration of HG reached 100 μM, it could significantly inhibit DOX-induced cytotoxicity (*p* < 0.05) (Figure 5C). Next, we evaluated whether the effect of HG was rooted in its inhibition of apoptosis. As anticipated, DOX observably increased the expression of cleaved caspase 3, by Western blot (*p* < 0.05) (Figures 5D, E). At concentrations less than 30 μM, HG had no effect on cell apoptosis (*p* > 0.05). Conversely, the expression of the cleaved caspase 3 protein obviously decreased as the concentration of HG increased above 30 µM (Figure 5D). To further test whether HG protects DOX-induced myocardial cell activity in a time-dependent manner, we added HG 30 min in advance and then DOX to stimulate cardiomyocytes for 48 h and found that HG could still protect the activity of DOX-induced cardiomyocytes (Supplementary Figure S2A). Thus, for the subsequent *in vitro* studies, the cells were treated with 3 µM DOX for 24 h with a concentration of 100 µM HG.

### Higenamine Alleviates Doxorubicin-Induced Neonatal Rat Ventricular Myocyte Oxidative Stress Injury *In Vitro*

**FIGURE 6:**
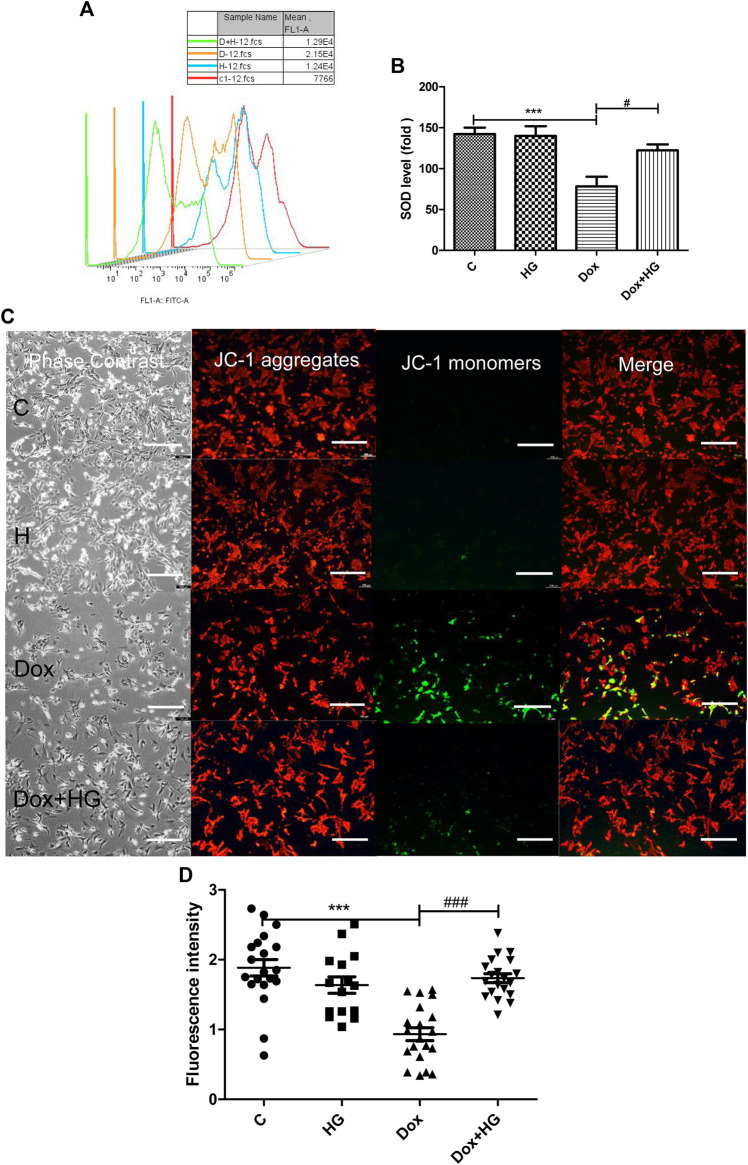
HG alleviated DOX-induced NRVM oxidative stress injury *in vitro*. **(A)** Content of ROS activity in NRVMs. **(B)** Content of SOD activity in NRVMs. **(C)** Mitochondrial membrane potential in cardiomyocytes were detected by using the JC-1 probe. The images were recorded using a microscope (Laika) at ×40 magnification. **(D)** Quantitative data of the JC-1 red-to-green fluorescence intensity ratio. The aforementioned experiments were repeated more than three times. All values are presented as mean ± SEM; statistical analysis was performed using Prism 8.0 one-way ANOVA. * *p* < 0.05 DOX vs Control C; # *p* < 0.05 DOX + HG vs DOX.

Oxidative stress injury is a central pathogenesis of DOX-induced myocardial apoptosis. Oxidative stress injury is primarily due to the imbalance between oxidative and antioxidant systems. Here, oxidative stress comprises an increase in reactive oxygen species and a decrease in antioxidant enzymes. We evaluated mean fluorescence intensity (MFI) to reflect the level of intracellular ROS. As shown in Figure 6A, DOX increased ROS production in NRVMs, while HG pretreatment can alleviate DOX-induced ROS production. In addition, we evaluated the level of superoxide dismutase (SOD) in NRVMs, as shown in Figure 6B. Here, HG could reverse the DOX-induced decrease in the expression of SOD.

The mitochondrial membrane is the premise of ATP production and necessary for maintaining mitochondrial function. The stability of mitochondrial membrane potential is conducive for maintaining normal physiological functions of cells. During oxidative stress, free radicals are not sufficiently eliminated from the cell, leading to the decline of mitochondrial membrane potential and the damage of mitochondrial function. JC-1 was used to detect the changes of mitochondrial membrane potential in NRVMs by fluorescence microscopy and analyzed by Image-Pro Plus. As shown in Figures 6C,D, DOX exposure changed JC-1 from a polymer to monomer, resulting in a decrease of red fluorescence and increase in green fluorescence, which suggested the loss of mitochondrial membrane potential in NRVMs after DOX treatment. Notably, pretreatment with HG can significantly improve the ratio of red to green fluorescence, indicating that HG can improve DOX-induced mitochondrial dysfunction.

Topoisomerase IIβ (Top 2β) is a protein that helps DNA in fixing topological difficulties and protects cells from being destroyed. DOX interacts with DNA and topoisomerase, forming the Top2–DOX–DNA complex, which increases double strand breakage, leading to cytotoxic effects (Tewey et al., 1984). As previously defined, Top 2β is an essential driver of DOX-induced cytotoxicity and DNA damage in cardiomyocytes (Zunino and Capranico, 1990). To explore whether HG is related to DOX-induced DNA damage by interfering with Top 2β, we detected Top 2β mRNA levels by qRT-PCR. We also observed that DOX administration reduced the Top 2β mRNA levels, but the levels of Top 2β were not reversed by HG intervention (Supplementary Figure 2B).

### Higenamine Blocked the AMPK Signal Pathway in Neonatal Rat Ventricular Myocytes and Mice

Previous studies have shown a similar structure of HG and catecholamine, which activates both β1- and β2-adrenergic receptors (AR). Our former studies reported that HG can reduce myocardial apoptosis induced by ischemia/reperfusion through activation of the β2/PI3K/Akt signaling pathway (Wu et al., 2016). We attempted to determine whether the inhibitory effect of HG on DOX-induced myocardial apoptosis is through the same pathway as that in ischemia/reperfusion-induced apoptosis. Here, we used specific β1-AR (CGP20712a), β2-AR (ICI118551), and PI3K inhibitors (LY294002). Interestingly, pretreatment with these three inhibitors did not change the protective effect of HG on DOX-induced NRVM apoptosis (Figure 7A), suggesting that the effect of HG on DOX-induced NRVM apoptosis is independent of the signaling pathway in ischemia/reperfusion disease.

**FIGURE 7A:**
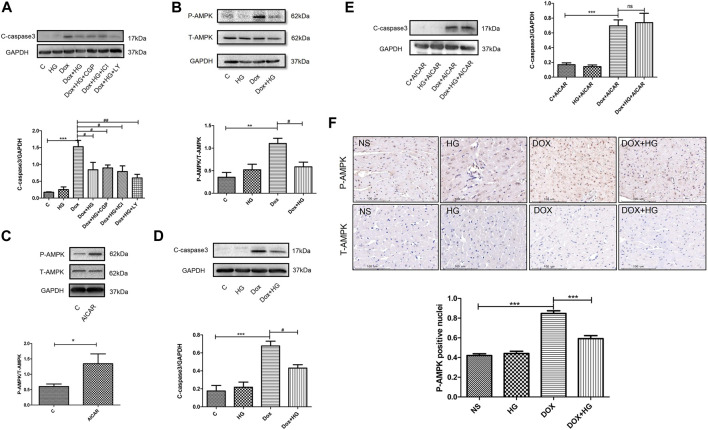
HG blocked the AMPK signal pathway in neonatal ventricular myocytes and in mice. **(A)** Effect of HG was not eliminated in the presence of CGP20712a (1 μM), ICI118551 (0.5 μM), and LY294002 (1 μM). **(B)** Western blot representative images and analysis of the expression of phosphorylated and total AMPK in neonatal ventricular cardiomyocytes. **(C)** Western blot representative images confirmed the efficacy of the agonist AICAR. **(D)** In the absence of agonist AICAR, HG could inhibit DOX-induced cardiomyocyte apoptosis. **(E)** AICAR blocked the inhibitory effect of HG on DOX-induced cardiomyocyte apoptosis. **(F)** Immunohistochemical staining of the phosphorylated and total AMPK in each group of 4 weeks and the phosphorylated AMPK-positive cell for quantitative analysis in mice hearts; scale bar: 100 μM. The aforementioned experiments were repeated more than three times. All values are presented as mean ± SEM; statistical analysis was performed using Prism 8.0 one-way ANOVA. * *p* < 0.05 DOX vs control C, AICAR vs control C, DOX vs NS; # *p* < 0.05 DOX + HG vs DOX; ns, *p* > 0.05 DOX + HG + AICAR vs DOX + AICAR. Animal numbers: NS, n = 8; HG, n = 7; DOX, n = 10; DOX + HG, n = 10.

**FIGURE 7B:**
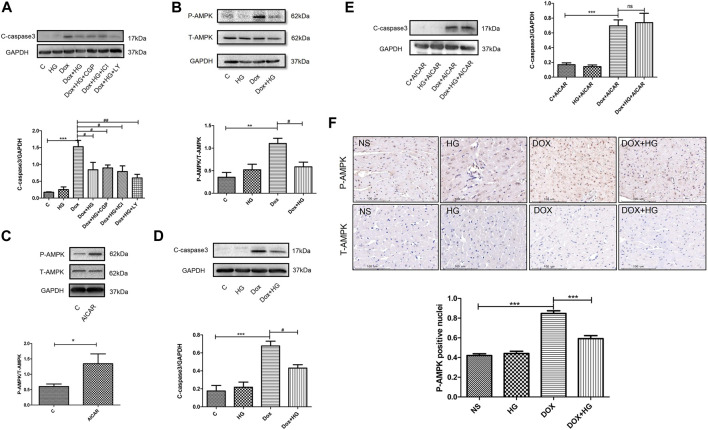
(Continued).

To explore the molecular mechanism of HG in DOX-induced NRVM apoptosis, we examined the AMPK signaling pathway, a typical pathway that participates in DOX-induced NRVM damage. Our results showed that the phosphorylation of AMPK was significantly increased in DOX-treated NRVMs, while HG significantly inhibited the phosphorylation of AMPK (Figure 7B). To further verify whether the AMPK signaling pathway contributed to the cardioprotective effect of HG, we used the AMPK-specific agonist, AICAR. As shown in Figure 7C, AICAR can increase the phosphorylation of AMPK, indicating the effectiveness of the agonist. In the absence of AMPK agonist AICAR, HG could inhibit DOX-induced NRVM apoptosis (Figure 7D). In the case of presence of AICAR, we found that the inhibitory effect of HG on DOX-induced NRVM apoptosis cold be blocked by AICAR (Figure 7E). Furthermore, the result of immunohistochemical staining showed that DOX treatment led to an increase in the level of phosphorylated AMPK-positive cells, and HG inhibited the number of phosphorylated AMPK-positive cells (Figure 7F). These results suggested that HG can inhibit DOX-induced NRVM apoptosis, and the protective effect of HG on the heart at least partially depends on the AMPK signaling pathway.

## Discussion

The cardiotoxic side effects of DOX, a typical anthracycline antitumor drug that has been prescribed for several decades around the world, have restricted the outcome for patients. At present, a few people have been successful in solving this problem. Dexrazoxane is the only drug that has been approved by the Food and Drug Administration (FDA) as a cardiac protective agent against DOX-induced cardiotoxicity (Kane et al., 2008). However, its use has been controversial due to its carcinogenic potential (Reichardt et al., 2018). Therefore, it is urgent to find new drugs to protect against DOX-induced cardiotoxicity. In Oriental Asia, aconite roots, a traditional Chinese medicine, has been commonly used to treat similar symptoms for thousands of years (Zhang et al., 2017). Higenamine (HG) is the main effective compound extracted from this Chinese herb. Recently, it has also been used as a common component in health products for fat degradation and sports performance in Europe and North America. Previous studies have shown that HG has positive inotropic and chronotropic effects on cardiomyocytes as well as antioxidant, antiapoptotic, and vasodilator effects (Zhang et al., 2014; Tariq and Aronow., 2015).

The aim of this study was to investigate the effect of HG on DOX-induced chronic cardiotoxicity. During our study, other groups reported the protective effect of 6-gingerol combined with HG on DOX-induced chronic heart injury (Chen et al., 2013; Wen et al., 2019; Wen et al., 2020). Herein, we provide further evidence that HG can prevent DOX-induced oxidative stress injury and improve cardiomyocyte apoptosis in neonatal rat cardiomyocytes *in vitro*. A DOX model was used to mimic clinical cardiotoxicity induced by chemotherapy drugs *in vivo*. We found that HG reduced DOX-induced myocardial atrophy and cardiomyocyte apoptosis and improved cardiac dysfunction and cardiac remodeling in mice. In general, our chronic cardiotoxicity mouse model and DOX cytotoxicity model demonstrated that HG could reduce DOX-induced cardiotoxicity.

The pathogenesis of DOX-induced cardiotoxicity has been a controversial and complex topic. Oxidative stress injury is one of the common causes of cell dysfunction and a vital factor in the pathogenesis of many diseases. Several studies have shown that DOX can accumulate in mitochondria, destroying the electron chain, increasing the content of ROS, and causing the imbalance of antioxidant and oxidative systems. This induces myocardial oxidative stress damage, further leading to myocardial cell apoptosis and ultimately causing cardiac dysfunction and even heart failure. In our study it also demonstrated that DOX can induce the increase of ROS, and the decrease of SOD content and mitochondrial membrane potential, all of which is consistent with previous research results. HG intervention can improve mitochondrial dysfunction, restore the mitochondrial membrane potential, promote the expression of superoxide dismutase, and reduce the production of ROS, indicating that HG can reduce DOX-induced oxidative stress injury.

Excessive production of reactive oxygen species can activate mitochondrial anion channels, further open mPTP channels, and reduce the mitochondrial membrane potential, all of which facilitate apoptosis triggered by the mitochondrial-dependent pathway (Sinha et al., 2013). In our *in vivo* study, TUNEL staining and Western blots showed that DOX could induce the increase of TUNEL-positive nuclei and the abundance of apoptosis protein cleaved caspase 3, indicating that DOX could induce cardiomyocyte apoptosis. Simultaneously, after HG intervention, the number of TUNEL-positive nuclei and the abundance of the apoptosis protein decreased significantly. These results indicated that HG could reduce cardiomyocyte apoptosis. In addition, we found that EF, FS, and SV were significantly improved after HG intervention, indicating that HG could improve DOX-induced cardiac dysfunction in mice.

Topoisomerase IIβ (Top 2β) is considered to be an important driver of the DOX-induced cardiotoxicity. Studies have found that DOX binds to Top 2β when entering the body, and a Top2–DOX–DNA complex is formed, which promotes ROS production, impairs mitochondrial function, and induces cardiomyocyte apoptosis (Tewey et al., 1984). In addition, dexrazoxane, the only drug that has been shown to prevent and treat DOX-induced cardiotoxicity, was reported to antagonize DOX-induced DNA damage by interfering with Top 2β (Lyu et al., 2007). Unfortunately, in our study, HG did not affect DOX-induced Top 2β expression.

The most well-known molecular target of HG is β-AR (Tsukiyama et al., 2009). Previous studies, including our own laboratory findings, have shown that HG is a β2-AR agonist in bronchorelaxation, and HG, through the activation of the β2/PI3K/Akt signaling pathway, inhibits cardiomyocyte apoptosis and protects myocardia from ischemia-reperfusion injury. Moreover, researchers have found that the heart-strengthening effect of HG is achieved through the β-adrenergic receptor pathway. In this study, we utilized the β1-AR agonist CGP20712a, β2-AR agonist ICI118551, and PI3K agonist LY294002 to investigate the mechanism of DOX-induced apoptosis inhibition by HG. Our studies found that HG could still protect against the DOX-induced apoptosis of cardiomyocytes. Notably, our results showed that HG inhibited DOX-induced cardiomyocyte apoptosis independent of the β-AR signaling pathway, suggesting that HG may have a new pharmacological target for anti-cardiomyocyte apoptosis induced by DOX.

Adenosine-activated protein kinase (AMPK) plays a key role in the regulation of biological energy metabolism. Previous studies have found that AMPK is a major regulator of lipid metabolism and glucose metabolism and is a hot topic in the study of diabetes and other metabolic diseases (Madhavi et al., 2019). In recent years, studies have revealed that the AMPK signaling pathway is also closely related to cardiovascular disease (Howell et al., 2011). Several studies support that the AMPK signaling pathway plays an important role in DOX-induced cardiac dysfunction (Feng et al., 2018). On the one hand, it is believed that activation of the AMPK signaling pathway can promote DOX-induced cardiomyocyte apoptosis (Liu et al., 2018). On the other hand, a few studies suggest that inhibition of the AMPK signaling pathway can play an antiapoptotic role in cardiomyocytes (Lv et al., 2012), which differs from the mainstream opinion of the AMPK signaling pathway. During our *in vitro* and *in vivo* study, DOX can significantly induce the phosphorylation of AMPK, while HG pretreatment can inhibit the phosphorylation of AMPK and protect cardiomyocytes from DOX-induced apoptosis. When AMPK agonist AICAR was added, the effect of HG disappeared. Therefore, the protective effect of HG may be achieved by inhibiting the AMPK signaling pathway. In the future, it is of great significance to study whether HG is related to the AMPK signaling pathway and the interaction between HG and AMPK signaling pathways.

## Conclusion

In this study, we found a new role of HG in tumor cardiopathy by inhibiting DOX-induced cardiomyocyte apoptosis. In addition, we demonstrated that this protective effect worked partially through the suppression of the AMPK signaling pathway. In the future, it is of great significance to study whether HG is related to the AMPK signaling pathway and the interaction between HG and AMPK signaling pathways.

## Data Availability

The original contributions presented in the study are included in the article/Supplementary Material, further inquiries can be directed to the corresponding authors.
