# Supplementary material for: Higenamine Attenuates Doxorubicin-Induced Cardiac Remodeling and Myocyte Apoptosis by Suppressing AMPK Activation
Source: Front Cell Dev Biol. 2022 May 5;10:809996. doi: 10.3389/fcell.2022.809996 (PMC9117701; doi:10.3389/fcell.2022.809996)
Supplement: Supplementary file 1 [file DataSheet1.PDF]

Supplemental Methods

Sirius red staining

Sirius red staining was performed as described previously. The mouse hearts were collected, fixed, embedded, and sectioned, according to the standard protocol. In short, heart sections were dewaxed with xylene and dehydrated with ethanol. The sections were incubated with the Weigert's haematoxylin for 8 min, washed with distilled water for 5 min, re-stained with Picro-sirius red solution for 45min, and finally washed with distilled water for 5 min. The sections were soaked in 75, 85, 95, and 100% ethanol for 2 min, then heart sections cleared in xylene and sealed with neutral resin.

Masson's trichrome staining

Masson's trichrome staining was following the manufacturer's instructions. The mouse hearts were collected, fixed, embedded, and sectioned, according to the standard protocol. In short, heart sections were dewaxed with xylene and dehydrated with ethanol. The sections were incubated with the Weigert's haematoxylin for 5 min, washed with distilled water for 5 min, 1% hydrochloric acid alcohol differentiation 5s, washed with distilled water for 5min, re-stained with Li Chunhong Acid Fuchsin Staining Solution for 5-10min, and rinse with distilled water quickly, treated phosphomolybdic acid aqueous solution for 3-5 min, directly dye with aniline blue solution for 5min, 1% glacial acetic acid treatment for 1min. The sections were soaked in 75, 85, 95, and 100% ethanol for 2 min, then heart sections cleared in xylene and sealed with neutral resin.

qRT-PCR analysis

RNA was extracted from isolated CMs via Trizol (Invitrogen) according to the manufacturer's instructions. For quantitative detection of mRNAs, reverse transcription of 100-500 ng total RNA prior to real-time qPCR was performed using the iScript Select cDNA synthesis kit (Bio-Rad), following the manufacturer's instructions. For each reaction, 1.0μL of cDNA was used in a 25μL reaction volume. Each reaction was performed in duplicate. GAPDH was used as housekeeping gene. The result is expressed as the ratio of target gene to GAPDH. The following primer pairs were used:

Rat BNP: 5′-TGACCAAGAACGATGGACACTAC-3′ (forward).
5′-CTGTGAACTCCCTCTTATGCTCC-3′ (reverse).
Rat GAPDH: 5′-TCAAGAAGGTGGTGAAGCAG-3′ (forward).
5′-TGGGAGTTGcTGTTGAAGTC-3′ (reverse).

Real-time qPCR was performed in a CFX96 Touch™ Real-Time PCR Detection System (Biorad, Hercules, USA) using specific primers and the iQ SYBR Green Mix (Bio-Rad) according to the manufacturer's protocol.

Elisa analysis

Elisa analysis according to the manufacturer's instructions**.** Remove the required lath

from the aluminum foil bag after balancing at room temperature for 10 minutes. Add sample or standard substance of different concentrations to the corresponding well at the rate of 100μL per well, empty Add 100μL universal dilution to white well. Cover with sealing plate film and incubate at 37℃ for 1 hour. Remove the plate, discard the liquid, and do not wash. Add raw directly to each well and cover with sealing plate membrane and incubate for 1 hour at 37℃. Discard liquid, add 300μL 1x solution to each well, stand for 1 min, then toss to wash Liquid, pat dry on absorbent paper, so repeat washing board 3 times. Add enzyme conjugate working solution 100μL to each well, cover with sealing plate membrane incubate at 37℃ for 30 minutes. Discard the liquid and wash the plate five times according to step 4. Add substrate (TMB)90μL to each well, cover with sealing plate film, and incubate at 37℃ for 15 minutes away from light. Take out plate, add stop solution 50μL to each well, within 15 minutes, at 450nm measure the OD value of each well at wavelength.

Supplemental Fig. 1

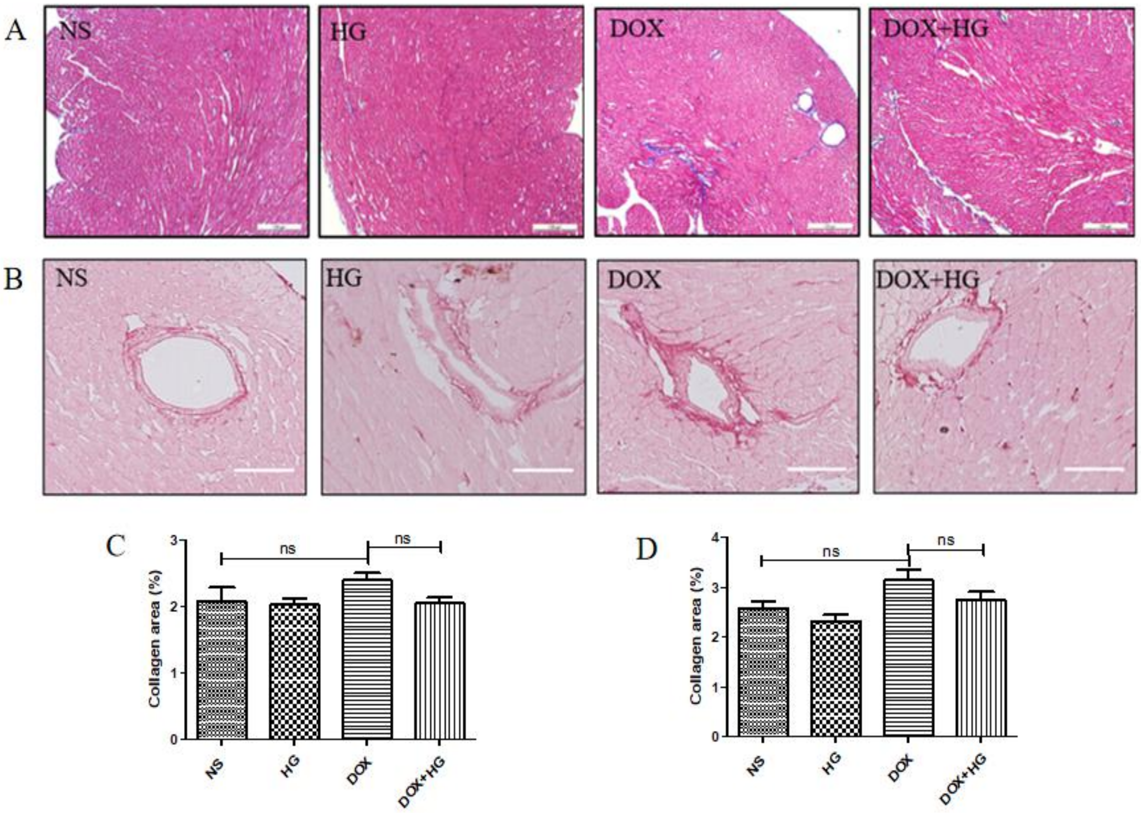

Supplemental Fig. 1. HG has no effect on preventing the development of cardiac fibrosis *in vivo*. (A) Schematic diagram of Masson's trichrome staining of the heart cross-sectional area for each group. Mice were injected with Normal saline (NS) or DOX(5mg/kg/3days) and systemically administrated with HG (10mg/kg/day) during DOX injection for 30 days. A scale bar:100μM. (B) Schematic diagram of Sirius red staining of the heart cross-sectional area for each group. A scale bar:25μM (C) Quantitative data of cardiac fibrosis expressed in mice with Masson's trichrome staining. (D) Quantitative data of cardiac fibrosis expressed in mice with Sirius red-staining. All values are presented as mean ± SEM, Statistical analysis was performed using Prism 8.0 one-way ANOVA. ns, P >0.05 DOX vs. NS; DOX + HG vs. DOX. Animal numbers: NS, n = 8; HG, n = 7; DOX, n=10; DOX + HG, n = 10.

Supplemental Fig. 2

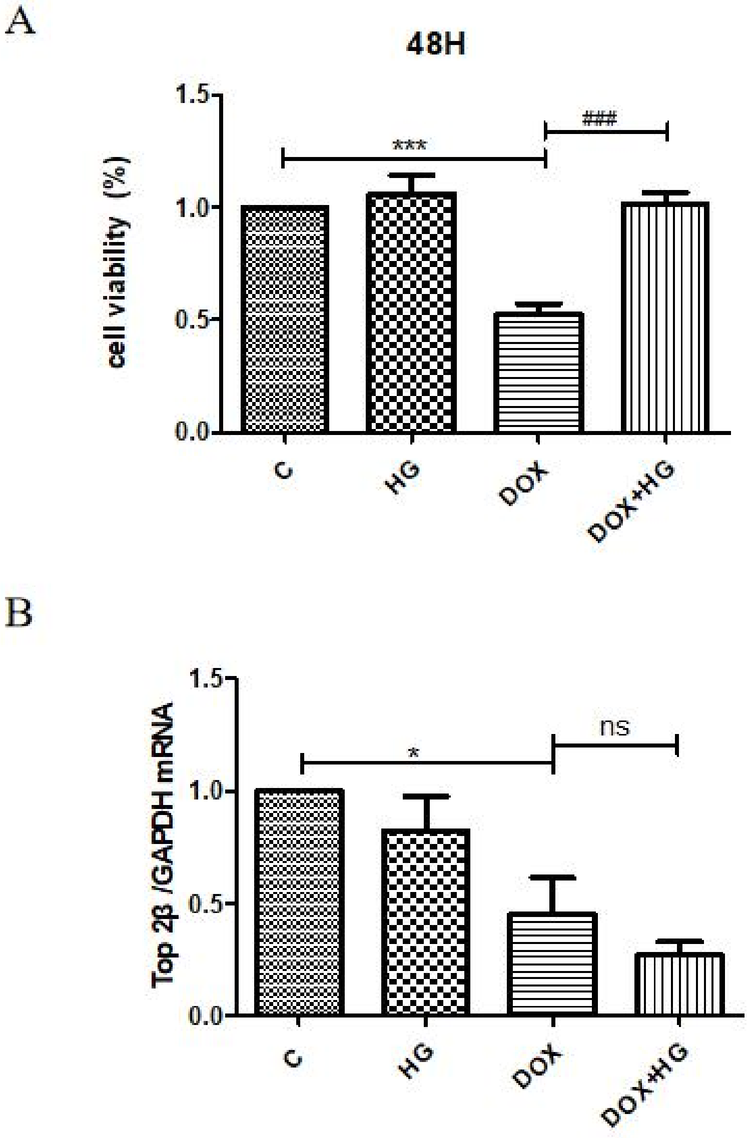

Supplemental Fig. 2. HG alleviated DOX-induced the decrease of myocardial cell activity *in vitro.* (A) Myocardial cells were pretreated with HG (100μM) for 30min after treatment with DOX(3 μ M) for 48h after detection by CCK8 reagent. (B) Real-time PCR analysis of Top 2β.The above experiments were repeated more than 3 times. All values are presented as mean ± SEM, Statistical analysis was performed using Prism 8.0 one-way ANOVA. *, $P < 0.05$ DOX vs. Control (C); ns, $P > 0.05$ DOX + HG vs. DOX.

Supplemental Fig. 3

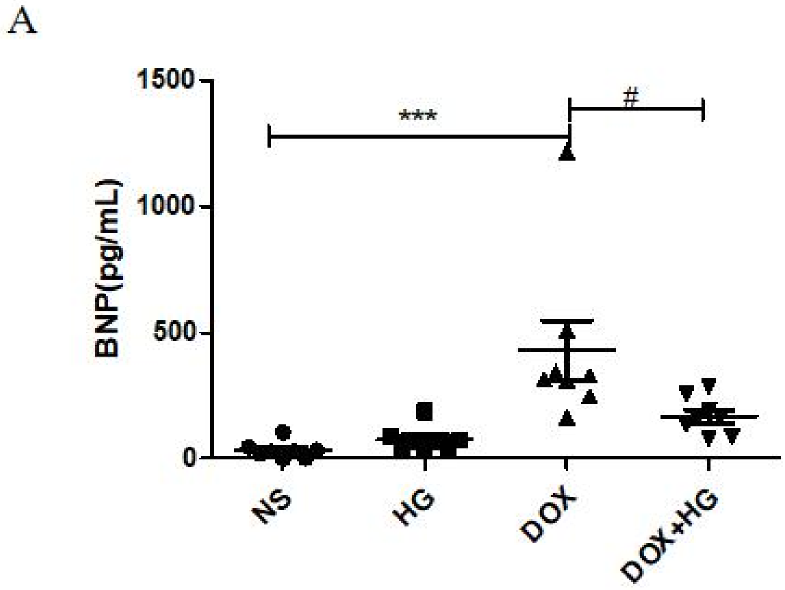

Supplemental Fig. 3. HG alleviated DOX-induced cardiac injury *in vivo*.
(A) BNP levels in serum detection by Elisa. All values are presented as mean ± SEM, Statistical analysis was performed using Prism 8.0 one-way ANOVA. *, $P < 0.05$ DOX vs. NS; #, $P < 0.05$ DOX + HG vs. DOX. Animal numbers: NS, n = 8; HG, n = 7; DOX, n=10; DOX + HG, n = 10.
